# Timing is key to providing modified assessments for students with specific learning difficulties

**DOI:** 10.1007/s40037-019-00553-4

**Published:** 2019-12-19

**Authors:** Christian P Gray, Steven A Burr

**Affiliations:** 1 Peninsula Medical School, University of Plymouth, PL4 8AA Plymouth, UK; 2 School of Clinical Medicine, Faculty of Medicine, University of Queensland, 4072 Brisbane, QLD Australia

**Keywords:** Specific learning difficulty, Learning disabilities, Modified assessment, Timing, Educational measurement

## Abstract

**Introduction:**

Medical students who are diagnosed with a specific learning difficulty (SpLD) will typically receive a reasonable adjustment within examinations in the form of modified assessment provision (MAP). This study investigated whether the timing of SpLD diagnosis and subsequent implementation of MAP has an impact on performance in applied medical knowledge multiple choice question (MCQ) assessments.

**Method:**

The MCQ performance of 108 students diagnosed with SpLD who received a MAP was monitored and compared with 1960 students who received an unmodified assessment, over 5 years of a medical program. Students who received a SpLD diagnosis in the latter years of the program were identified as not receiving a MAP in assessments prior to diagnosis.

**Results:**

Differences were found between declaration and diagnosis, with 44.4% of students who declared and 48.1% who did not declare subsequently receiving a diagnosis. Students with SpLD who receive a MAP increase their applied medical knowledge assessment performance, although there is a delay of up to a year for this impact to reach significance.

**Conclusion:**

Early diagnosis of SpLD is necessary to ensure the intended benefit is received from MAP.

## What this paper adds

Although modified assignment provisions are universally applied across a range of assessments, there is little or no evidence that they provide benefit for students with specific learning difficulties. The use of retrospective assessment data from medical students who received a specific learning difficulty diagnosis late in their program provides a unique opportunity to examine the impact of a modified assessment provision on medical knowledge assessment. Students who received a specific learning difficulty diagnosis and subsequent modified assessment provision significantly increased their applied medical knowledge assessment performance. The timing of diagnosis and provision of the modified assessment provision was particularly important.

## Introduction

A specific learning difficulty (SpLD) is defined as an impairment within learning that is unexpected given other learning abilities. Areas that may be impaired include, but are not limited to attention, concentration, reasoning, understanding, memory and coordination [1, 2]. It is challenging, however, to find an international consensus within assessment guidelines or literature, with the condition defined as a specific learning difficulty, disability or disorder depending on national diagnosis guidelines [1, 3, 4]. To maintain consistency the term SpLD will be used within this paper. In 2009, 4.1% of medical students disclosed a disability, which included SpLDs [5]. Dyslexia is the most common SpLD affecting 3–10% of the general population in the UK [1, 6] and 10–15% in the US [7]. Currently up to 2% of students entering UK medical schools are diagnosed with dyslexia, twice what it was 10 years ago [8]. SpLD may be associated with differential attainment within medical education. Affected students often receive a reasonable adjustment (or accommodation) within examinations in the form of modified assessment provision (MAP) in order to ‘level the playing field’ [3, 9]. In the UK, the General Medical Council states that ‘students with a wide range of disabilities or health conditions can achieve the set standards of knowledge, skills, attitudes and behaviour’ [10]. There is similar guidance from other international medical professional bodies including Australia, United States and Canada [11–13]. In many countries it is unlawful to discriminate against the education of a student because of their disability. This is in line with the Equality Act 2010 within the UK [14], Disability Discrimination Act 1992 in Australia [15] and ADA Amendments Act of 2008 in the USA [16]. Therefore, a student with a disability can be accepted into medical school, as long as they can be provided with reasonable adjustments to support their performance, which do not compromise the assessment of clinical competence standards [17]. The most common form of MAP is extra time to allow for slower abilities in reading, comprehension and/or writing [9], but can also include formats facilitating enlarged size, altered font, or colour combinations [18]. The General Medical Council published a comprehensive list of types of MAP used within medical schools [19]; however, a survey of UK medical schools demonstrated variation in their implementation [20]. This may be related to different types of assessment or indicate some confusion between schools on the types of MAP that may be appropriate for different types of assessment [20]. The General Medical Council guidelines also state ‘only those students who are fit to practice as doctors should be allowed to complete the curriculum and gain provisional registration’ [10]. This inconsistency in guidelines between ‘fit for study’ and ‘fit for practice’ may make it challenging for a school to reconcile MAP during education, with a desire to empower students towards the workplace, where fewer reasonable adjustments are typically available.

There is currently little research into the performance of students with SpLD in medical education [21]. Two UK medical schools have examined students with SpLDs as part of a larger study on assessment using multiple choice question (MCQ) based progress testing. They showed that there was no significant difference between students with SpLD who received a MAP and students without disabilities [9, 22]. These studies examined a snapshot of medical knowledge assessment performances and were limited to suggesting that SpLD students do not perform significantly differently because of their MAP. Given that all SpLD students within these studies received a MAP they cannot identify if students required the MAP or would have performed equally well without it. Furthermore, no considerations were made within these studies regarding the timing of SpLD diagnosis or MAP. Currently, although MAPs are universally applied across assessments, there is little or no evidence that they provide benefit within medical examinations [21]. Students receiving a diagnosis of SpLD late in the course of their studies provide an opportunity to assess the benefit of a MAP in SpLD students. The study focuses on the impact of a MAP on progress test performance in which students receive extra time. Other forms of assessment including OSCEs were not included because students do not receive additional time. This is consistent with previous studies which reported no difference in OSCE performance in students with or without dyslexia [9]. The aims of the current study were to further elucidate:

1. whether students with SpLD benefit from a MAP; and
2. whether the timing of a SpLD diagnosis and subsequent implementation of MAP affects summative performance.

## Methodology

### Participants

**Fig. 1:**
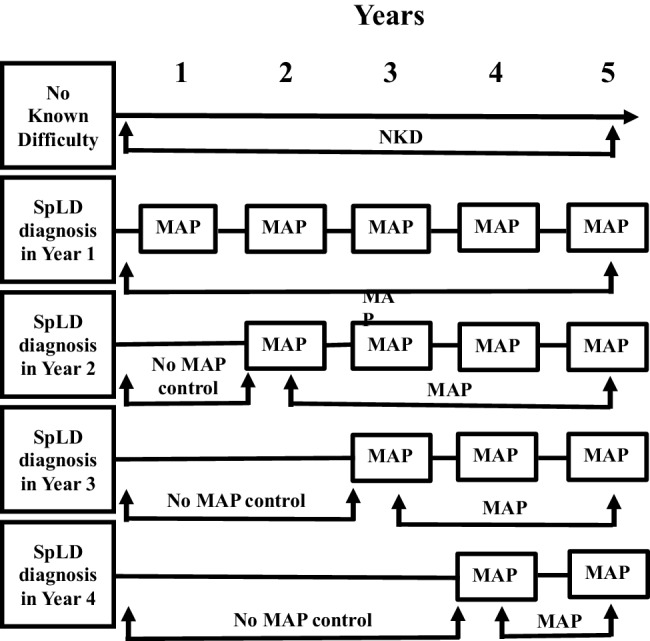
Diagnosis of SpLD and timing of MAP in progress test. A conceptual model was constructed to demonstrate when a student would receive a modified assessment provision (MAP) or standard assessment (a no MAP control) based on year of SpLD diagnosis. Students with no known difficulty received a standard assessment

This study explores the effectiveness of MAP for 108 students diagnosed with a SpLD within Peninsula Medical School between 2002 and 2016. Declaration of a SpLD through self-assessment to the UK Universities and Colleges Admissions Service was used to identify the level of disclosure prior to admission to medical school. SpLD within this study refers to a specific learning difficulty such as dyslexia, dyspraxia or attention deficit hyperactivity disorder (code 51, Higher Education Statistics Agency, UK). Students were diagnosed through university Disability Services with an assessment by a psychologist or psychiatrist. SpLD students received a MAP recommendation of an extra 20 minutes per hour of examination and their exams were printed on buff paper. Students who received additional or alternative MAP, for example rest breaks, were excluded from the study. The date from which a student received a MAP was used to identify progress test performance pre (−1) and post (+1) provision of MAP. Students who received a SpLD diagnosis in the latter years of the program were identified as not receiving a MAP in assessments prior to diagnosis. Given that these students were later diagnosed with a SpLD they provided a ‘no MAP control group’ within assessments prior to diagnosis. Fig. 1 shows a conceptual model of SpLD diagnosis and MAP. This figure indicates ‘MAP’ and ‘No MAP control’ groups based on when a student received a SpLD diagnosis.

### Performance in progress test multiple choice questions

Peninsula Medical School uses a progress test, a type of MCQ-based testing used to assess longitudinal growth in applied medical knowledge assessment performance [22]. Assessment of students with SpLD by progress testing has been described previously [23, 24]. Briefly, the progress test is conducted 4 times a year, with the same test taken by all students in all years of the program. The test consists of 125 single best answer questions, each question comprising a clinical vignette, a choice of five possible answers and a ‘don’t know’ option. Each question is assessed with +1 mark for a correct answer, −0.25 for an incorrect answer and 0 for a ‘don’t know’ response. Comparisons of scores were made within, as opposed to across, student groups to account for differences in the level of knowledge between year groups.

### Statistical analysis

Progress test performances were retrospectively analyzed for students who were diagnosed with a SpLD in years 1–4 between 2002–2016. Students who were diagnosed with a SpLD in year 5 were excluded from this analysis because of limited numbers. First attempt student performances before and after MAP were compared using paired t-tests. Comparisons between MAP arrangements were made using a Bonferroni’s multiple comparison test.

## Results

### Incidence of SpLD diagnosis and the impact of disclosure

**Fig. 2:**
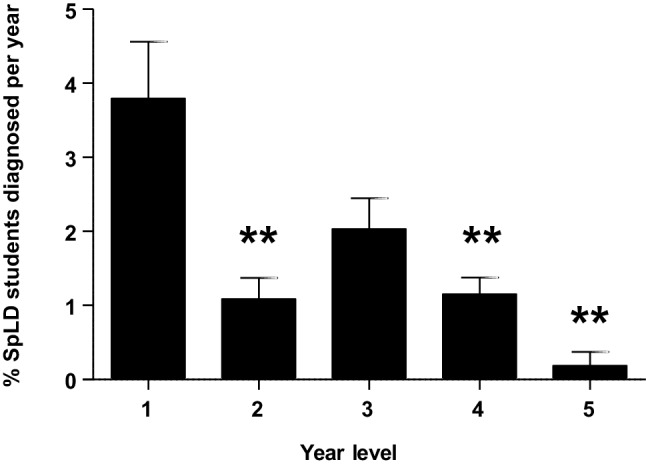
Incidence of SpLD diagnosis by year of study. Data display frequency of SpLD diagnosis by study year. Significantly increased in year 1 and year 3 compared with years 2, 4 and 5 (*p* < 0.001, Bonferroni’s multiple comparison test)

A total of 2068 students were enrolled onto the medical program between 2002 and 2016. Over this period 108 students were diagnosed with a SpLD (1.6 ± 0.3% of the total students received a diagnosis per year). As shown in Fig. 2 there was a significantly higher number of students who received a diagnosis within their first year of study (*p* < 0.001, Bonferroni’s multiple comparison test) (46, 3.8 ± 0.8% year 1 students diagnosed per year). The number of students diagnosed rose throughout the program, with year 3 being the next highest incidence of new disclosure (26, 2.0 ± 0.4% year 3 students diagnosed per year). There was no profound difference in the types of SpLD diagnosis between year 1 and years 2–5 (data not shown). Tab. 1 indicates that of the 46 students diagnosed with a SpLD in year 1, only 39 disclosed a SpLD on their entry application to medicine. Of the remaining students who were diagnosed with a SpLD, four students also disclosed a non-learning disability or condition. In addition, three students did not disclose on entry, but subsequently received a diagnosis within the first year of study. Not all students who did disclose a SpLD on their entry application to medicine were diagnosed within the first year. Six, two and one student(s) were subsequently diagnosed in years 2, 3 and 4 respectively. Tab. 1 summarizes the number of students receiving a MAP across all years. Of the students who received a MAP, 44.4% (48/108) disclosed a SpLD on their entry application to medicine. A further 7.4% (8/108) disclosed a non-learning disability or condition, and 48.1% (52/108) did not disclose this on their entry application to medicine. Of students who disclosed a SpLD on entry to the program, only 53.3% (48/90) subsequently requested a MAP. To our knowledge no student who requested a MAP had been refused.

**Table 1:** Disclosure and diagnosis of specific learning difficulty

Year	Diagnosed with an SpLD^a^	Disclosed an SpLD^b^	Report no known difficulty^b^	Disclosed a non-learning disability or condition^b^
1	46	39	3	4
2	19	6	11	2
3	26	2	22	2
4	15	1	14	0
5	2	0	2	0
Modified^c^	108	48	52	8
Standard^d^	0	42	1850	68

^a^Students were diagnosed through Disability Services

^b^Declaration to the UK Universities and Colleges Admissions Service

^c^Students received a MAP recommendation of an extra 20 minutes per hour of examination

^d^Students examined under standard conditions

### Importance of early SpLD diagnosis and MAP

**Fig. 3:**
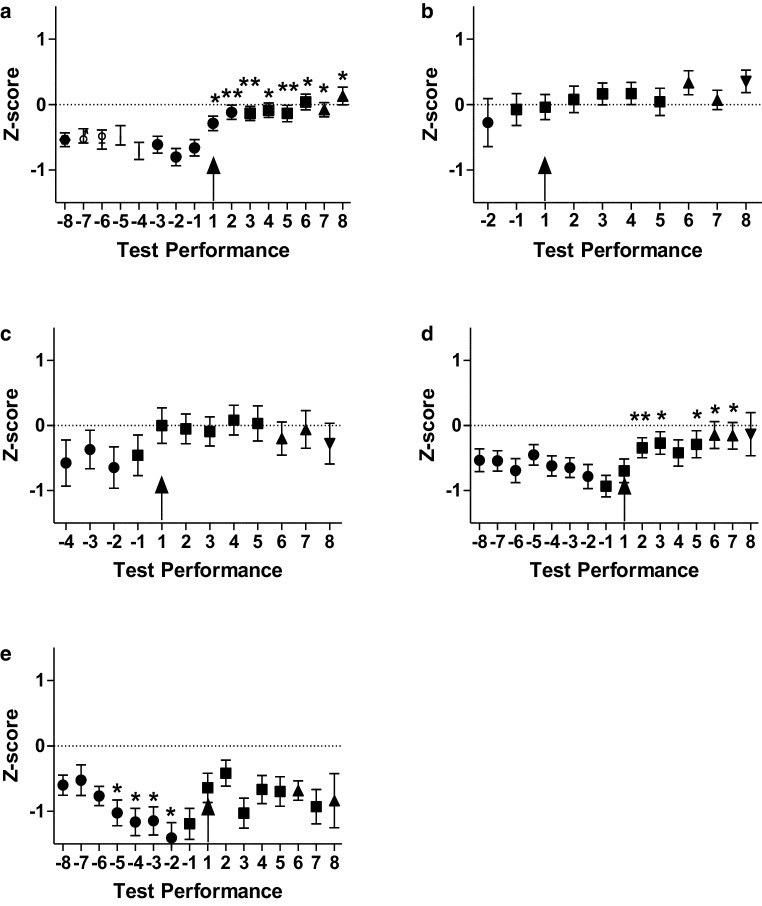
Enhancement of medical knowledge assessment performance in students with SpLD receiving a MAP. Z‑scores were analyzed from progress test performances for students with SpLD who received their first MAP (**a**) across all years 1–4, (**b**) year 1, (**c**) year 2, (**d**) year 3; (**e**) year 4. The arrow indicates the test in which students received their first MAP. Introduction of the MAP afforded significant enhancement of medical knowledge assessment performance when all years were combined or when students received their first MAP in year 3 (test performance pre (−1) vs post (+) MAP (* *p* < 0.05, ** *p* < 0.001, paired T‑test))

Medical knowledge assessment performance was analyzed based on when students with SpLD first received their MAP. Fig. 3a demonstrates that when data for all year levels are combined, students with SpLD significantly enhanced their medical knowledge assessment performance through MAP (*p* < 0.05, paired t‑test). There was no significant difference, however, in medical knowledge assessment performance for students with SpLD who received their first MAP in years 1 or 2 (Fig. 3b, c). Fig. 3d demonstrated that students diagnosed with a SpLD in year 3, upon receiving a MAP, significantly enhanced their medical knowledge assessment performance for up to seven tests post MAP (*p* < 0.05, paired t‑test). A MAP for students diagnosed in year 4 did not significantly change their medical knowledge assessment performance (Fig. 3e). Prior to diagnosis of a SpLD there was a significant decrease in medical knowledge assessment performance up to four tests prior (*p* < 0.05, paired t‑test) but no significant change post MAP.

**Fig. 4:**
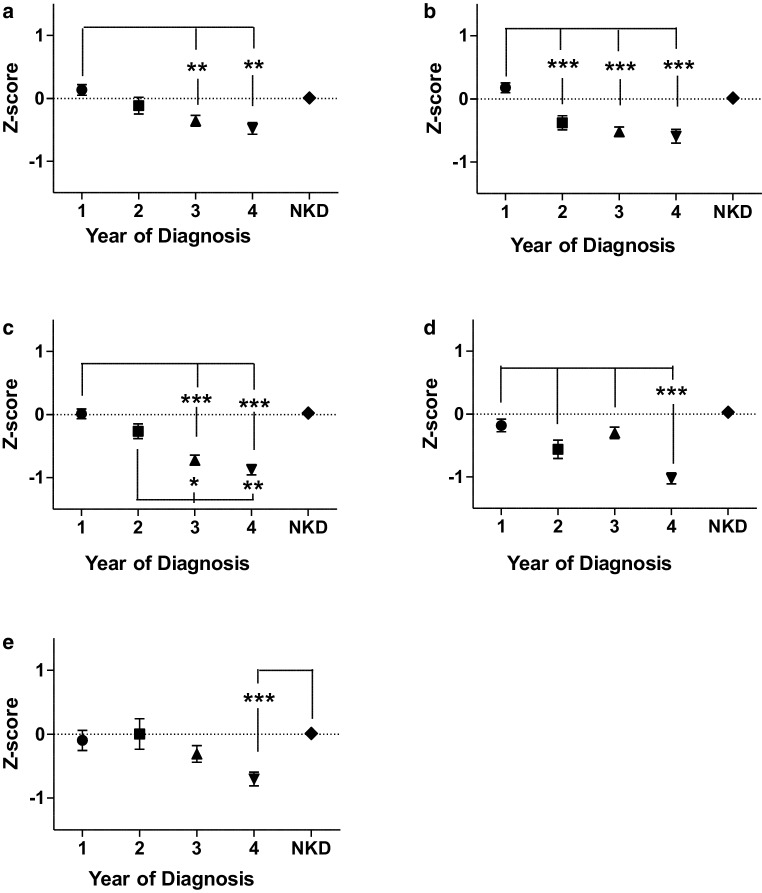
Positive effect of MAP is dependent on time of SpLD diagnosis. Z‑scores were analyzed from progress test performances for students with SpLD in (**a**) year 1, (**b**) year 2, (**c**) year 3, (**d**) year 4 and (**e**) year 5. ‘NKD’ refers to no known difficulty and ‘Year of diagnosis’ refers to the year level in which students were diagnosed and received their first MAP. Students who were diagnosed in year 1 demonstrated significant enhancement of medical knowledge assessment performance compared with students diagnosed in other years within the second year of assessment (*p* < 0.0001, Bonferroni’s multiple comparison test). Students who were diagnosed in year 2 demonstrated significant enhancement of medical knowledge assessment performance compared with students diagnosed in years 3 or 4 within the third year of assessment (*p* < 0.05 and *p* < 0.001 respectively, Bonferroni’s multiple comparison test). Students who were diagnosed in year 3 demonstrated significant enhancement of medical knowledge assessment performance compared with students diagnosed in year 4 within the fourth year of assessment (*p* < 0.0001 respectively, Bonferroni’s multiple comparison test). In year 5, there was only a significant difference between students who were diagnosed in year 4 and no known difficulty (*p* < 0.0001 respectively, Bonferroni’s multiple comparison test)

Fig. 4 demonstrates the importance of early MAP in enhancing students’ medical knowledge assessment performance. The assessment data suggest that a student with SpLD requires over a year to significantly increase applied medical knowledge assessment performance. In Fig. 4a, students with SpLD who were diagnosed and received a MAP showed increased medical knowledge assessment performance in year 1 compared with students with SpLD who were diagnosed in years 3 or 4 (*p* < 0.001, Bonferroni’s multiple comparison test). Within the second year of assessment the students with SpLD who received a diagnosis and MAP from year 1, showed significantly increased performance compared with all years (*p* < 0.0001, Bonferroni’s multiple comparison test) (Fig. 3b). Students with SpLD who were diagnosed and received a MAP in the 2nd year showed significantly improved performance within the 3rd year of assessment compared with students with SpLD diagnosed in years 3 or 4 (*p* < 0.05, Bonferroni’s multiple comparison test) (Fig. 4c). Students with SpLD who were diagnosed and received a MAP in their 3rd year showed significantly improved performance within the 4th year of assessment compared with students with SpLD diagnosed in year 4 (*p* < 0.001, Bonferroni’s multiple comparison test) (Fig. 4d). Fig. 4e shows that by year 5, there were no significant differences between medical knowledge assessment performance in students with SpLD who were diagnosed and received a MAP in years 1–3. There was a significant decrease, however, between medical knowledge assessment performance in students who were newly diagnosed in year 4 and students with no known difficulty (*p* < 0.001, Bonferroni’s multiple comparison test).

## Discussion

Students with SpLD may find MCQ questions more difficult compared with other forms of examination because of the reading demands involved, as well as possible difficulties in visual processing [1]. Within this study students who received a diagnosis of dyslexia, dyspraxia or attention deficit hyperactivity disorder were declared to have a SpLD and received a MAP of additional time when undertaking progress tests. Diagnosis of a SpLD can be complex and often requires a variety of evaluations, performed by a member of a regulated profession [8]. The level of impairment due to SpLD is thought to manifest over a wide spectrum and not all students may meet the clinical threshold to achieve a diagnosis [1]. This may explain in part the differences between students who initially disclose a SpLD, but subsequently do not receive a diagnosis. Disclosure might also confer an advantage for pre-entry exams where evidence of a diagnosis may not be required [25], or conversely access to MAP in pre-entry exams did not meet student expectations and so a diagnosis may not have been pursued. Some students may also not wish to disclose a SpLD for fear of discrimination at or after entry into medical school [3, 26]. This may explain why some students who initially disclosed a SpLD on entry to medicine waited until later in the program to seek and receive a diagnosis, perhaps after receptiveness had been explored. Due to student anonymity, we are however unable to identify how many students do not receive a diagnosis. The move towards a supportive and diverse culture within the school is recommended in which students are encouraged to be empathetic towards people with disabilities. This may allow students to feel more comfortable in disclosing their own SpLD [3].

In year 3 of the program there was a significant increase in SpLD diagnosis (*p* < 0.001). This suggests that some students may initially be unaware of their SpLD and seek a diagnosis after experiencing the pressure of the curriculum and the need to adapt to multiple learning styles [23]. Such students are more likely to be found within the medical profession, possessing high levels of intelligence and ‘milder’ specific impairments [8]. They have developed strategies to overcome many of the obstacles associated with their impaired learning and have already developed compensatory mechanisms to pass examinations [11]. These students may not have been considered to have a SpLD prior to entry to higher education given their high level of academic achievement. Alternatively, they may have found it difficult to gain a definitive diagnosis [7, 27]. Within Peninsula Medical School, students are often asked to reflect on the possibility of a SpLD diagnosis through the support from disability services [7, 27] or in response to difficulties within assessment leading to a holistic remediation intervention [24]. Therefore, it should not be assumed that the students who do not declare or who are diagnosed later in program would have a different level of impairment. Caution should also be used in considering self-disclosure of a SpLD upon application to medical school to identify the number of students with SpLDs or the level of support required. It is clearly important to differentiate diagnosis from disclosure when establishing the impact of SpLD on performance. In this study we have shown evidence that students with a SpLD receiving a MAP significantly increase their applied medical knowledge assessment performance. The use of retrospective assessment data from students who received a SpLD diagnosis late in the program, provides a unique opportunity to examine a ‘no MAP control group’ prior to diagnosis, as SpLD is always present at a constant level throughout life [2]. The timing of diagnosis and provision of modified assessment was found to be particularly important. Although students with a SpLD will receive a MAP immediately after diagnosis, a significant increase in medical knowledge assessment performance may not be seen for up to a year after intervention. This suggests that students may take time to adapt to the provisions provided within the MAP. The greatest improvement in medical knowledge assessment performance after MAP was observed in year 3 students, which through the pressure of moving to a more clinical environment may have prompted them to seek a SpLD diagnosis [23]. This is also consistent with the significant decrease (*p* < 0.05) in medical knowledge assessment performance in students who did not receive a diagnosis of a SpLD until year 4. Although these students subsequently received a MAP there was a significant decrease in medical knowledge assessment performance in year 5 compared with students without disabilities (*p* < 0.001). The delayed impact of the MAP may influence the validity of other studies that pool data from students with SpLD without considering the point of diagnosis. The interpretation that all students with SpLD are not adversely affected may have been masked by differences in medical knowledge performance depending on the length of time since they received their MAP intervention. This is particularly evident in the study by Ricketts et al. which examined a snapshot of medical knowledge performance for students with SpLD within the Peninsula Medical School [23]. Likewise, combining performances of students with SpLD across all year levels should also be discouraged given the potential impact of differences in the level of student knowledge.

It is important to recognize that MAP is not the only support students receive upon diagnosis of SpLD. Students may also receive extra support through specialist equipment, resources and additional academic support. Academic support may include specific guidance in essay writing, time management, exam preparation techniques and other study skills [28]. In addition, the SpLD diagnosis itself may give students insight into how to compensate for their weaknesses, as well as possible areas of strength to explore, for instance, problem-solving skills and the ability to think multi-dimensionally [29]. This may help explain why students’ performances were significantly poor until after they received a diagnosis and MAP. The impact of this additional support could not be examined within this study given that all students were given a MAP upon diagnosis with a SpLD. A follow-up qualitative study is recommended to understand students’ perception of the diagnosis and support for their SpLD.

Thus the data in this study suggest there are differences in performance depending on the interval between diagnosis and summative assessment. Early diagnosis of SpLD is necessary to ensure the intended benefit is received from MAP. This is further evidenced by the end of medical school (year 5), in which students who were diagnosed with a SpLD and received a MAP up to two years prior attained a similar attainment to students without disabilities.
